# Supplementary material for: Highly efficient gene transfer in the mouse gut microbiota is enabled by the Incl2 conjugative plasmid TP114
Source: Commun Biol. 2020 Sep 22;3:523. doi: 10.1038/s42003-020-01253-0 (PMC7508951; doi:10.1038/s42003-020-01253-0)
Supplement: Supplementary file 1 — Supplementary Information [file 42003_2020_1253_MOESM1_ESM.pdf]

Supplementary Materials

Highly efficient gene transfer in the mouse gut microbiota is enabled by the Incl$_2$ conjugative plasmid TP114

Kevin Neil, Nancy Allard, Frédéric Grenier, Vincent Burrus and Sébastien Rodrigue*.

*Correspondence to: Sebastien.Rodrigue@USherbrooke.ca

This file includes:

- Supplementary Methods
- Supplementary Figs. 1 to 6
- Supplementary Tables 1 to 3
- Supplementary References 1 to 8

Supplementary Methods

Construction of *E. coli* KN01Δ*dapA*. A DAP auxotrophic variant was obtained by deleting the *dapA* gene in EcN[1] by recombineering using pSIM6. To generate a DAP auxotrophic strain, the *aph-IIIa* resistance cassette of pKD4 was amplified by PCR with added homology for the regions flanking *dapA*. A second PCR round on the purified PCR product then allowed to increase the length of homology. EcN containing an activated pSIM6 was electroporated with the purified PCR product, followed by kanamycin resistance selection. Insertion of the cassette and deletion of *dapA* were then verified by PCR with corresponding primers (Supplementary Table 3). After confirmation of *dapA* gene locus replacement by the pKD4 resistance cassette, the strain was cured from pSIM6 by heat-shock at 42°C for 1 hour followed by overnight incubation at 37°C. The culture was then streaked on selective plates to screen for ampicillin sensitive clones. Then, the strain was transformed with pE-FLP to knock-out the resistance cassette inserted in the genome as described previously[2]. pE-FLP plasmid was cured by heat-shock following the same procedure as for pSIM6. Next, the insert from pGRG36-SmSp was added in the genome of EcNΔ*dapA* strain to obtain KN01Δ*dapA*.

Generation of rifampicin and nalidixic resistant mutants of EcN. Plasmid TP123, RIP71a and R1 cannot be tested using our standard set of 3 strains (KN01, KN02, KN03) because they harbor the resistance genes used to select donor and recipient pairs (Supplementary Tables 1 and 4). As such, an additional resistance was needed to test the transfer of these plasmids. We opted for rifampicin and nalidixic acid resistances, as both are chromosomally encoded and easy to obtain. To generate spontaneous resistant variants of KN01, KN01Δ*dapA* and KN02, each strain was first started from frozen stock and allowed to grow overnight. Then, 200 µL of each culture was transferred in LB broth containing 0.5X of the working concentration of either rifampicin or

nalidixic acid and incubated overnight at 37°C. The resulting cultures were then streaked on plates containing the working concentration of rifampicin or nalidixic acid and plates were incubated at 37°C overnight. A single colony was isolated, creating KN01Rf^R, KN01Δ*dapA*Rf^R, and KN02Nx^R. For all three plasmids, KN01Δ*dapA*Rf^R was next used as the donor bacterium for *in vitro* conjugation experiment and KN01Rf^R as the donor for *in situ* conjugation experiment while the recipient strain was always KN02Nx^R.

Mice inoculum preparation. Two days prior to gavage, a frozen stock of the strain(s) used for colonization was streaked onto MacConkey selective plates and incubated overnight at 37°C. The next day, several colonies were inoculated in selective LB broth at 37°C. Three to four hours prior to mice oral challenge, strain(s) were sub-cultured again with a large inoculum (200 µL or 500 µL) in 20 mL selective LB broth and incubated at 37°C until an OD_{600nm} of 0.6 ± 0.1 was reached. The cells were then washed once in PBS and concentrated in a volume equivalent to 6.0 OD_{600nm} according to initial OD_{600nm} in LB. A volume of 100 µL of the cell suspension was force fed to each mouse (corresponding to ~$1x10^8$ CFU). Part of the inoculum was plated and analyzed for CFU counts. Cell concentration ranged from 10^8 to 10^9 CFU/mL for all experiments.

Feces sampling. Collection tubes were prepared prior to the experiment by aseptically adding 500 µL of PBS and a single 0.2 mm glass bead to a 1.5 mL tube. Tubes were weighted before and after sampling to normalize CFU by sample weight. Then, samples were homogenized in a FastPrep-24 (MP) beads beater for 1 minute at maximum speed. The homogenates were centrifuged at 500 x *g* for 30 seconds to prevent possible pipetting of larger debris. The centrifugation step did not show a significant impact on retrieved CFU. The samples were then serially diluted 1/10 in sterile PBS from 10^0 to 10^{-7} of the initial concentration and 2.5 µL of each dilution was spotted on selective MacConkey plates in technical duplicates. For each experiment,

total *Enterobacteriaceae* clearance was also followed on MacConkey plates without antibiotics as a control for streptomycin treatment.

Mice dissection and EcN colonization pattern assessment. On the last day of the experiment, the mice were anesthetized with isoflurane, sacrificed by cervical dislocation and dissected to extract the duodenum, jejunum, ileum, caecum, ascending colon and/or descending colon. To distinguish the parts of the intestine, the first 3 cm of small intestine attached to the stomach were considered to be duodenum, the 6 central cm the jejunum and the 6 last cm (closest to the caecum) the ileum. The ascending and descending colons were the exact halves of the colon. Two spaced quarters of each section and the longitudinal half of the caecum were sampled for CFU analysis. Since the caecum is a large and distinct structure of the mouse intestine, and EcN was shown to strongly colonize the caecum (Figure 2a), this region was chosen as a representative part of the intestine to study colonization.

TP114 sequencing. TP114 was acquired from DSMZ (DSM-4246) and transferred from *E. coli* K-12 J53-2 by conjugation into *E. coli* MG1655Nx^R. The resulting strain was then grown at 37°C in selective LB broth to obtain enough DNA for sequencing. An Illumina library was prepared using the QIAseq FX Library kit (Qiagen) from size-selected genomic DNA fragments of approximately 400 to 600 base pair (bp). The Illumina library was sequenced on a MiSeq instrument using paired-end reads of 300 bp to assemble longer composite reads covering the entire insert[3]. A MinION (Oxford Nanopore Technologies, UK) sequencing library was also prepared using 1.5 µg of high-molecular weight genomic DNA and the R9 Nanopore sequencing kit (SQK-NSK007, Oxford Nanopore Technologies, UK). Illumina sequencing reads were assembled with the Roche gsAssembler v2.6 either *de novo* or using reference sequences from other conjugative plasmids from the $IncI_2$ family (R721, AP002527.1; pChi7122, FR851304;

pRM12761, CP007134.1; pSLy21, NZ_CP016405.1). Large *de novo* and reference contigs were then manually assembled and scaffolded with high-quality MinION reads using BLASTn v2.8.1. Finally, 10 regions of 1.5 kb were selected based on lower read coverage and were re-sequenced by Sanger sequencing to confirm the assembly with corresponding primers (Supplementary Table 3). The resulting circular sequence was submitted to the RAST v2.0 annotation server[4], and a total of 92 open reading frames were predicted. The annotation was then adjusted to name homologous genes consistently between TP114 and the reference $IncI_2$ plasmid R721 (GenBank: AP002527.1).

Sequencing and annotation of other conjugative plasmids. Plasmids were acquired from various sources (Supplementary Table 1). Upon arrival, strains containing the plasmids were grown overnight in LB broth at 37°C to obtain enough material for DNA extraction. Illumina libraries were prepared using NEBNext Ultra II DNA Library Prep Kit selecting DNA fragment of 150-300 bp. The Illumina libraries were sequenced on a NextSeq 500 instrument using paired-end reads of 75 bp to assemble longer composite reads covering the entire insert[3]. Illumina sequencing reads were assembled with the Roche gsAssembler v2.6 either *de novo* or using reference sequences when available. Contigs mapping on *E. coli* chromosome were screened out of the assembly to purify reads covering the plasmid DNA. The resulting circular sequences or large contigs were submitted to the RAST v2.0 annotation server[4]. The resulting DNA assembly was submitted to Genbank when significant differences were found with the reference sequence or when the sequence for the plasmid was missing from Genbank.

HDTM libraries sequencing. For each sample, a 1.5 mL frozen stock aliquot of mutant library was thawed on ice for 15 minutes. The aliquot was centrifuged, and cells were resuspended in 300 µL of cell lysis buffer from the Quick gDNA Miniprep kit (ZymoResearch). DNA was

fragmented using a Bioruptor UCD-200 sonication system (Diagenode) for 12 cycles of 30 seconds ON, 30 seconds OFF at 4°C. After fragmentation, the Quick gDNA Miniprep kit protocol for cell suspension was performed and DNA was eluted in 50 μL of molecular grade water. 10 μg of the eluted DNA was end-repaired using End-repair Mix HC (Enzymatics) followed by DNA purification with AMPure DNA XP magnetic beads (Agencourt). Purified DNA was then adenylated by TaqB (Enzymatics) supplemented with dATP for 30 minutes at 68°C and purified again with AMPure DNA XP beads (Agencourt). Nextera adaptator B was generated by annealing 2 oligonucleotides: 5'-PO_4-CTGTCTCTTATACACATCTCCGAGCCCACGAGAC-InvdT-3' and 5'-CAAGCAGAAGACGGCATACGAGATTCGCCTTAGTCTCGTGGGCTCGGAGATGTGTA TAAGAGACAGT-3' together. Annealing was performed by heating 40 μM of each oligonucleotide in annealing buffer (10 mM Tris NaCl pH 7.5, 50 mM NaCl) to 98°C and then slowly decreasing the temperature by 0.1°C each 10 seconds until 4°C was reached. Nextera adapter B was ligated using T4 DNA ligase (Enzymatics) overnight at 16°C. DNA was purified again using DNA Ampure XP beads (Agencourt) and barcoding was performed in a qPCR machine using Veraseq DNA polymerase (Enzymatics). Amplification reaction was stopped at the end of the exponential phase. DNA was purified again and quantified using Quant-it PicoGreen dsDNA assay (Thermo Fisher). Quality and size distribution of the amplified mutant library was assessed on an Agilent 2100 Bioanalyzer instrument using a High Sensitivity DNA Chip. Mutant libraries were then pooled and sequenced by Illumina using the Nextera technology.

Deletion of *pilS* in TP114. An FRT flanked *cat* gene was amplified from pKD3 (Supplementary Table 3). The PCR product was used to delete *pilS* in TP114 by recombineering. The

recombinant clones were then screened using appropriate primers (Supplementary Table 3). Cells were then heat-shocked as described in the recombineering section to get rid of the pSIM6 plasmid. Then, to eliminate the *cat* gene from TP114Δ*pilS*::*cat*, cells were transformed with pE-FLP. Chloramphenicol sensitive clones of TP114Δ*pilS* were confirmed for *cat* loss by PCR and transferred to *E. coli* strain KN01. The ability of the wild-type and *pilS* mutant versions of TP114 to transfer from *E. coli* KN01 to *E. coli* KN03 was assayed under solid, liquid (static), liquid (shaking) and *in situ* conditions.

Induction of *pilS* expression. Plasmid pPilS was constructed by amplifying the *pilS* gene from TP114, $oriV_{p15A}$-*araC*-P_{BAD} from pBAD30 and *cat* from pSB1C3 using primers listed in Supplementary Table 3 and joining the amplification products by Gibson assembly. Plasmid pPilS was then transformed into KN01 + TP114Δ*pilS* for complementation studies. The *pilS* gene is under the regulation of AraC[5], its expression is therefore inducible by arabinose. For *pilS* complementation experiments, donor and recipient strains were grown overnight at 37°C. Two hours before conjugation, arabinose was added to the donor strain cultures at a final concentration of 1% w/v. Then, OD_{600nm} of each culture was measured and cells were washed in LB + 1% arabinose then resuspended in a volume equivalent to 40 OD_{600nm} in LB + 1% arabinose. A volume of 2.5 µL of the donor and recipient strain were then mixed together and deposited on an LB + 1% arabinose plate for solid conjugation or mixed with 195 µL of pre-warmed LB + 1 % arabinose for both liquid static and liquid shaking conjugations. Conjugation experiments in all conditions were then incubated at 37°C for 2 hours. Additionally, liquid shaking conjugation were placed on a rotary agitator. After incubation, the conjugation experiments were serially diluted 1/10 and plated on selective media for CFU analysis of the donor, recipient, and transconjugant strains.

Supplementary Figure 1: **Specific features of strains used in the conjugation mouse model.**

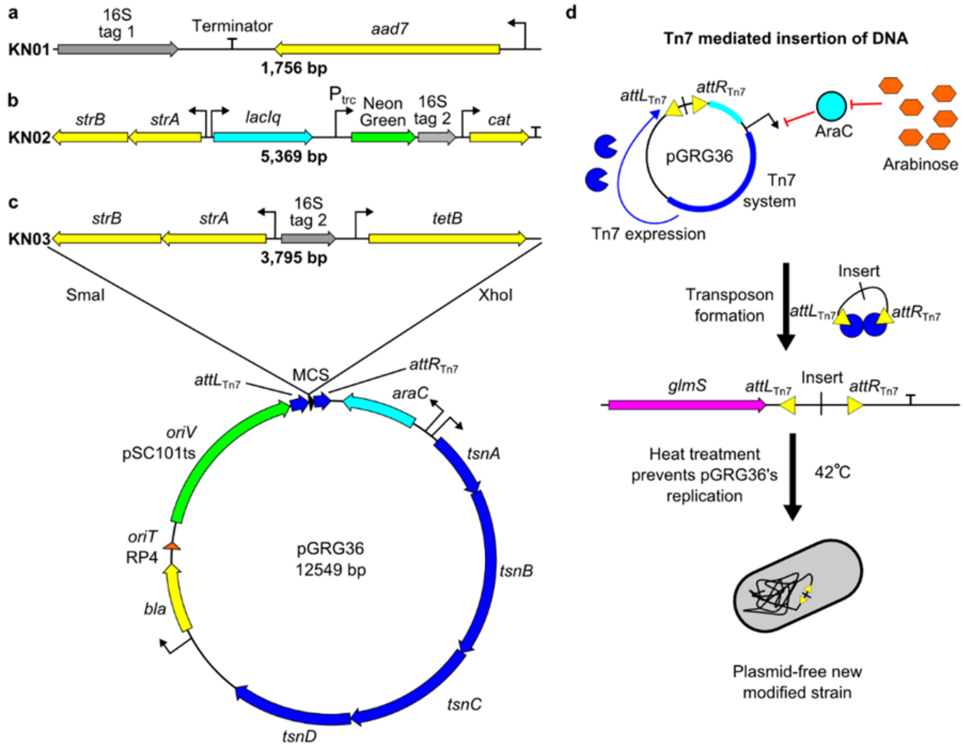

Antibiotic resistance inserts were designed to allow quantification of conjugation. Each insert was added into EcN genome using site-specific recombination, creating KN01 (**a**), KN02 (**b**), and KN03 (**c**). Schematic representation of the insertion strategy for the antibiotic resistance markers in EcN genome using an arabinose inducible Tn7 plasmid system (**d**).

Supplementary Figure 2. EcN colonization optimization.

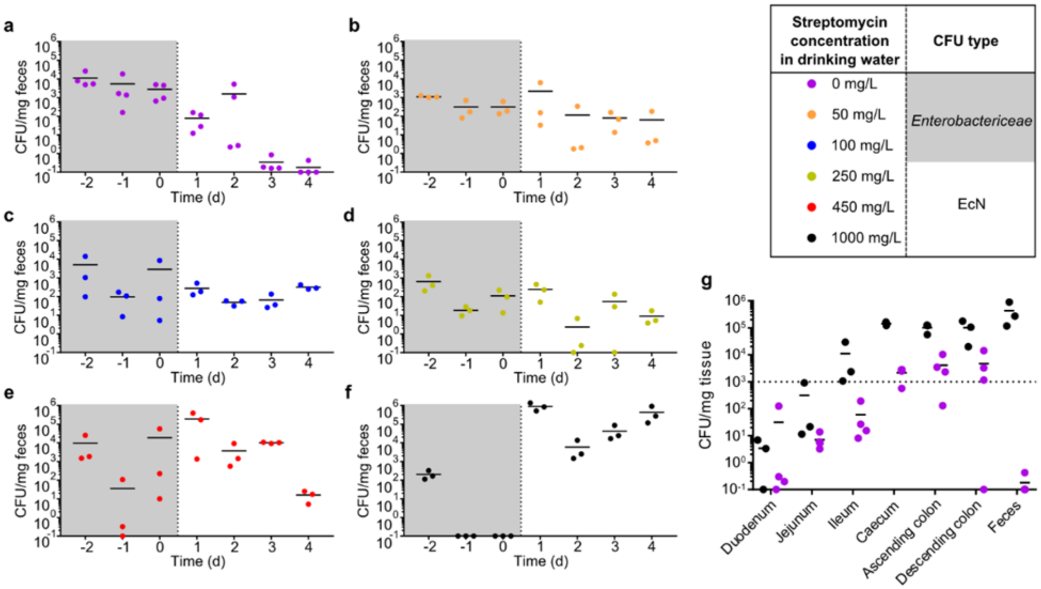

Enterobacteriaceae depletion (gray area) and EcN colonization (white area) followed by CFU counts from feces of mice treated with 0 mg/L (n=4, biologically independent mice) (**a**), 50 mg/L (n=3, biologically independent mice) (**b**), 100 mg/L (n=3, biologically independent mice) (**c**), 250 mg/L (n=3, biologically independent mice) (**d**), 450 mg/L (n=3, biologically independent mice) (**e**), and 1000 mg/L (n=3, biologically independent mice) (**f**) of streptomycin in drinking water starting at day -2. **g**, Colonization levels of EcN KN01 in various sections of the intestinal tract for Sm-treated (black) or untreated (purple) mice 3 days after introduction in mice (n=4, biologically independent mice).

Supplementary Figure 3: Transfer rates of 13 plasmids in the mouse intestinal tract.

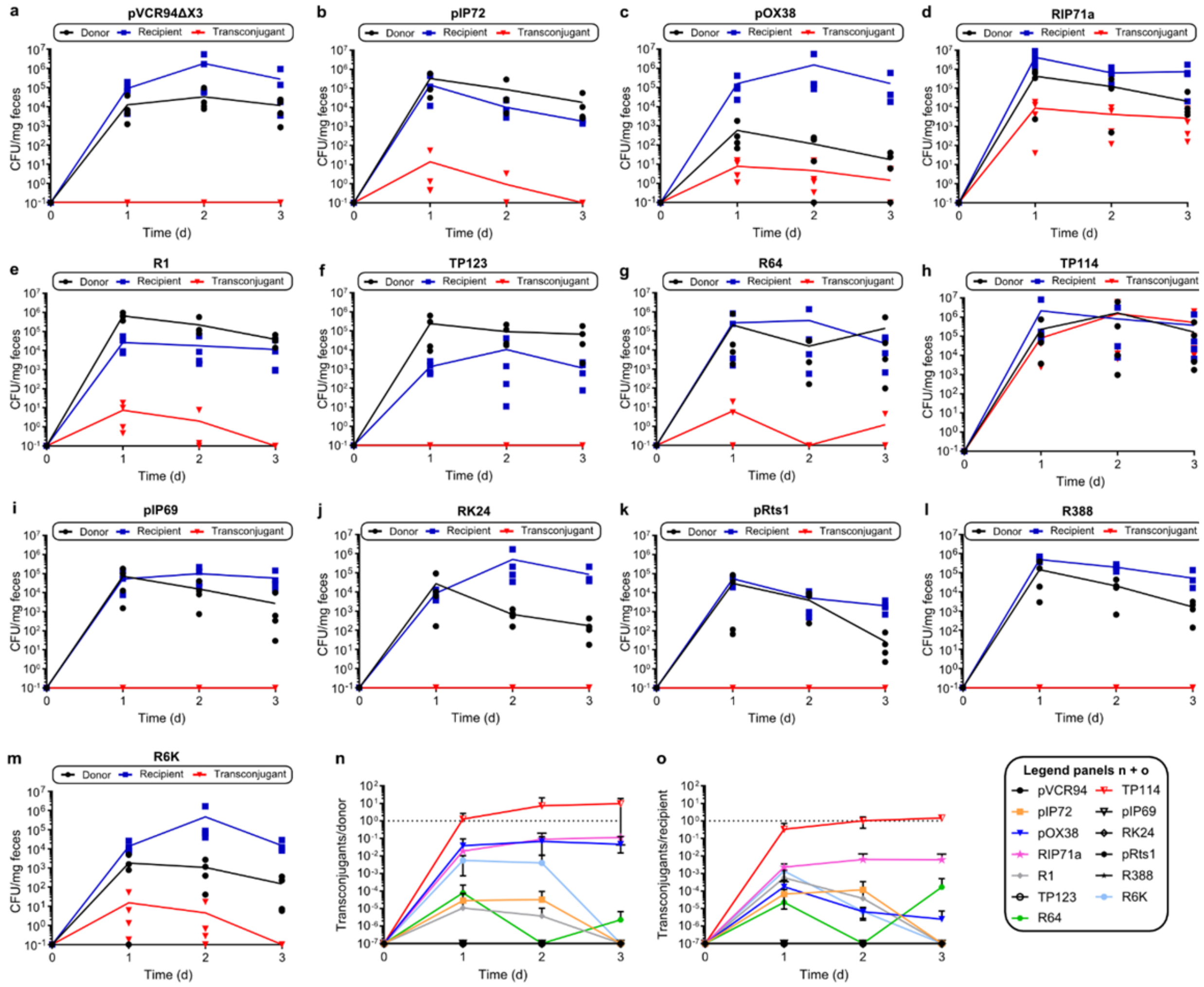

Raw CFU counts of the donor strain (KN01), the recipient strain (KN02 or KN03) and transconjugants in feces collected on day 0, 1, 2 and 3 after the introduction of KN01 for plasmid pVCR94ΔX3 (**a**), pIP72 (**b**), pOX38 (**c**), RIP71a (**d**), R1 (**e**), TP123 (**f**), R64 (**g**), TP114 (**h**), pIP69 (**i**), RK24 (**j**), pRts1 (**k**), R388 (**l**), and R6K (**m**) (n=4). Calculated transfer rates per donor (**n**) and per recipient (**o**) are shown (n=4, biologically independent mice).

Supplementary Figure 4: Analysis of TP114 gene essentiality by high-density transposon mutagenesis (HDTM).

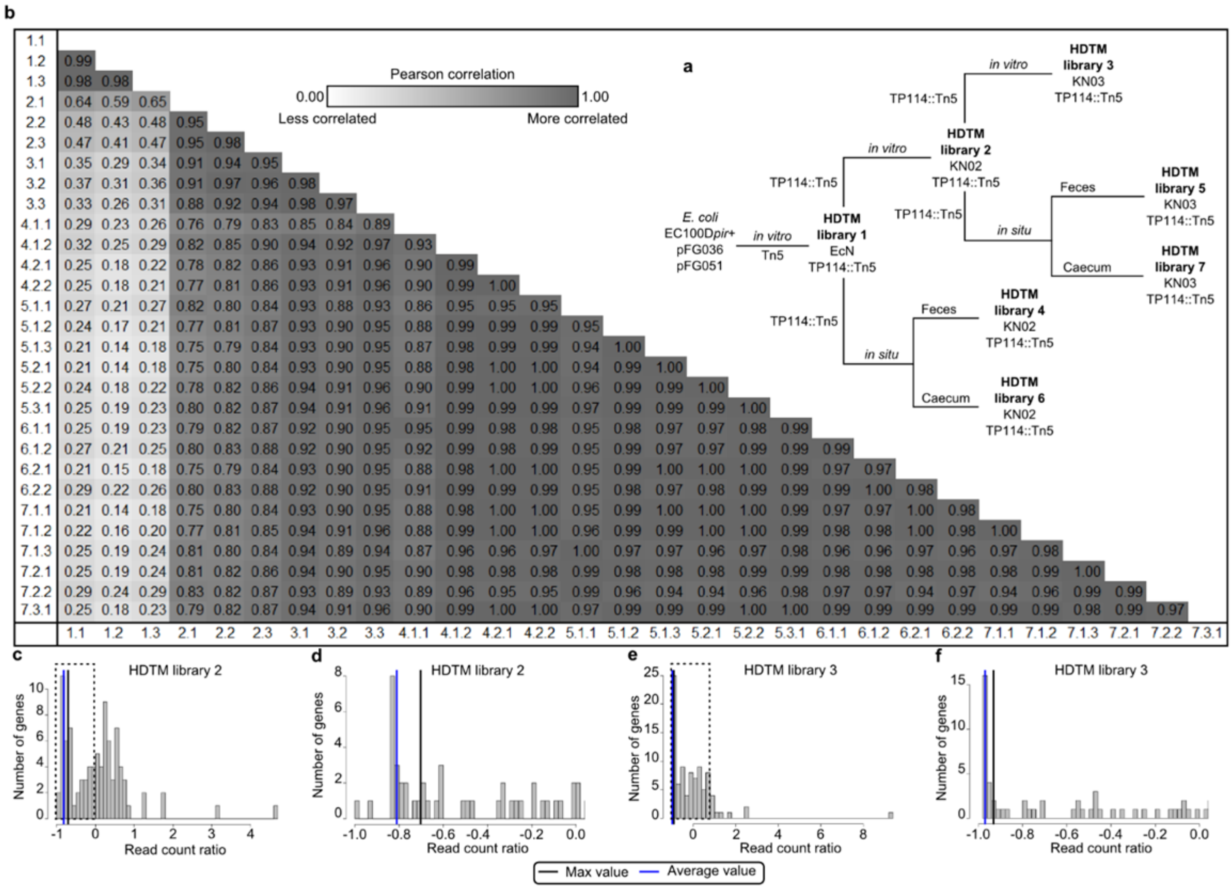

a, Initial mutant library 1 was selected for mutants able to perform *in vitro* or *in situ* conjugation, generating HDTM library 2 to 7. **b,** Pearson correlations between HDTM libraries annotated in a X.Y.Z format where X=HDTM library, Y=library replicate and Z=Mouse when applicable. Gene essentiality was determined by calculating the difference in read counts between HDTM library 1 and test conditions. Essentiality thresholds (max value, black line) were set using a core set of predicted essential genes, as exemplified with HDTM library 2 (**c**) and 3 (**e**). Panel **d** and **f** are a zoom of the framed section of panel **c** and **e**, respectively.

Supplementary Figure 5: Sequence homology between TP114 and other plasmids of the IncI family.

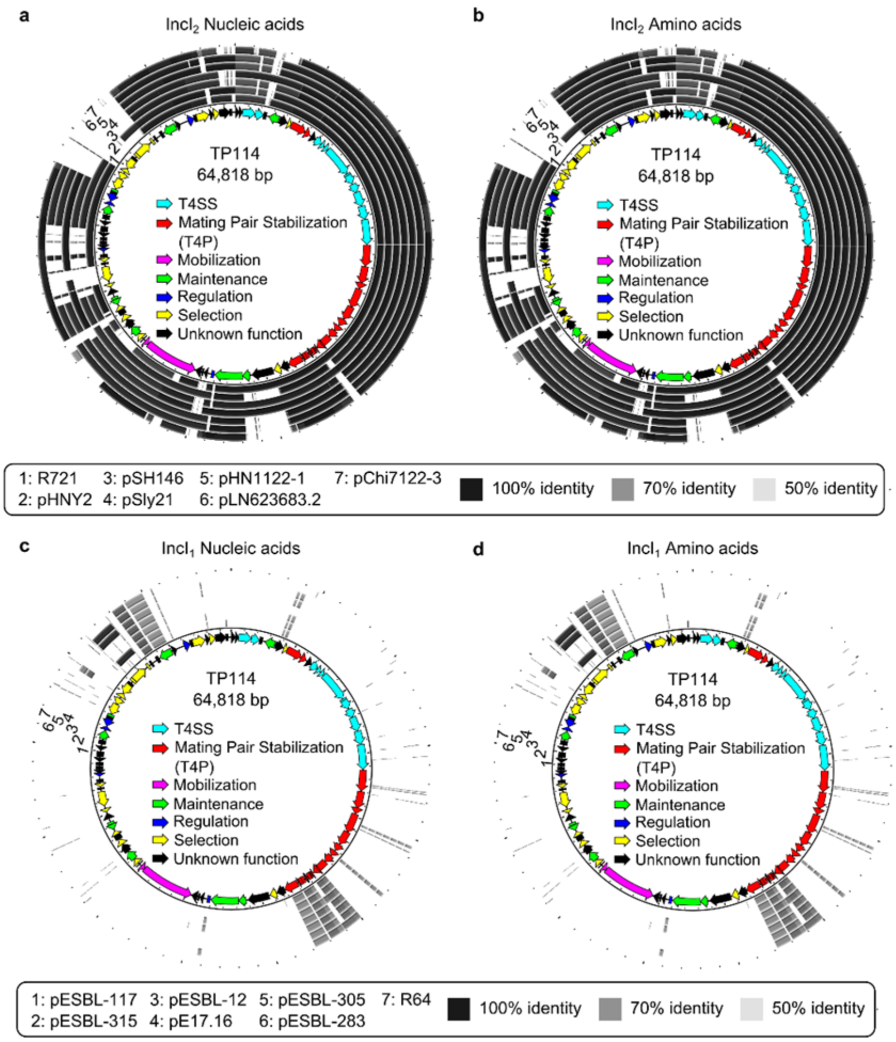

Sequence homology was evaluated using BRIGG, a BLAST-like program that shows sequence identity in circular pattern. Sequence identity threshold were set at 100%, 70% and 50% for all analysis. TP114's sequence was first compared to seven members of the IncI$_2$ subfamily based on the nucleic acid sequences (**a**), and the amino acid sequences of its coding genes (**b**). Gene conservation among IncI$_2$ plasmids is also compiled in Supplementary Data 1. TP114 was then compared to seven members of the IncI$_1$ subfamily based on the nucleic acid sequences (**c**), and

the amino acid sequences of its coding genes (**d**). Numbers on homology rings correspond to the plasmids in the legend with 1 being the innermost ring and 7 being the outermost ring.

Supplementary Figure 6: TP114 gene essentiality and conservation among IncI$_2$ plasmid family members.

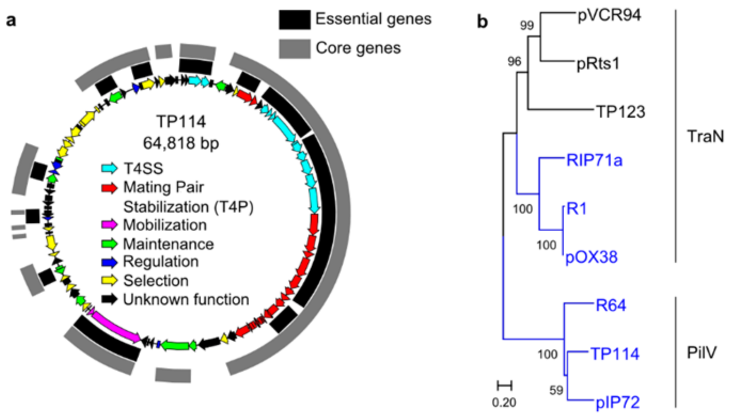

a, the TP114 plasmid map displayed in the innermost circle starts at TP114-01 at the top of the circle and continues clockwise with TP114-02 up until TP114-92. Gene essentiality in any HDTM condition as experimentally tested in Figure 3 is highlighted in black while core IncI$_2$ genes as determined by comparative genomics analysis shown in Supplementary Figure 5 are highlighted in gray. **b**, phylogenetic analysis of known adhesin genes involved in mating pair stabilization. Conjugative plasmids that transferred *in situ* are shown in blue.

Supplementary Table 1: Conjugative Plasmids used in this study.

Name	Inc group	MOB group	MPF group	*In situ* conjugation	Resistance	Isolated in	Size (bp)	CDS count	Genbank	Source
pVCR94ΔX3	C	H12	F	-	Km, Su	*Vibrio cholerae*	121,195	152	KF551948.1	V. Burrus lab
pIP72	B/O	P7	I	+	Km	*Escherichia coli*	Unknown	140	MN612051	CRBIP19.42
pOX38	FI	F12	F	+	Sp, Tc, Su	*Escherichia coli*	59,705	76	MF370216.1	L. Frost lab
RIP71a	FII	F12	F	+	Ap, Tc, Cm, Sm, Sp	*Escherichia coli*	Unknown	112	MN626601	CRBIP19.47
R1	FII	F12	F	+	Km, Cm, Su, Sp	*Salmonella enterica*	97,566	135	KY749247.1	CRBIP19.58
TP123	HI	H11	F	-	Sm, Cm, Su, Sp	*Salmonella typhi*	Unknown	222	MN6266042	CIP TP123
R64	I1	P12	I	+	Sm, Tc	*Salmonella typhimurium*	120,826	150	NC_005014.1	CRBIP19.30
TP114	I2	P6	T	+	Km	*Escherichia coli*	64,818	92	MF521836.1	DSM-4246
pIP69	L/M	P13	I	-	Ap, Km, Tc	*Salmonella enterica*	70,500	111	MN626603	CIP pIP69
RK24	P1α	P11	T	-	Ap, Tc	*Escherichia coli*	60,096	70	BN000925.1	DSM-3876
pRts1	T	H12	F	-	Km, Sp	*Proteus vulgaris*	Unknown	162	MN626604	CRBIP19.43
R388	W	F11	T	-	Su, Tm	*Escherichia coli*	33,913	43	NC_028464	DSM-5189
R6K	X2	P3	T	+	Ap, Sm	*Escherichia coli*	39,872	52	NZ_LT827129.1	DSM-4245

Supplementary Table 2: High Density Transposon Mutagenesis Tnseq results.

Library. Replicate. Mouse	Expected number of reads	Raw reads	% pFG051	Trimmed reads	% trimmed aligned on EcN	Aligned on TP114	% trimmed aligned on TP114	Insertion sites	Insertion sites per Kilobase	Average distance between sites (bp)
1.1	5.20E+07	5.19E+07	4.43	4.81E+07	79.02%	5.55E+06	11.53%	11385	175.65	5.69
1.2	5.20E+07	6.37E+07	8.00	6.02E+07	75.05%	6.59E+06	10.95%	10873	167.75	5.96
1.3	5.20E+07	4.96E+07	8.59	4.68E+07	76.05%	5.25E+06	11.21%	12710	196.09	5.10
2.1	6.20E+05	2.38E+06	6.68	2.25E+06	65.79%	5.35E+05	23.77%	11470	176.96	5.65
2.2	6.20E+05	1.48E+06	0.33	1.36E+06	18.60%	1.07E+06	79.06%	9745	150.34	6.65
2.3	6.20E+05	6.93E+05	0.30	6.53E+05	13.60%	5.50E+05	84.21%	8929	137.75	7.26
3.1	6.20E+05	9.29E+05	0.21	8.49E+05	3.52%	7.78E+05	91.71%	5050	77.91	12.84
3.2	6.20E+05	7.89E+05	1.09	7.23E+05	11.61%	6.03E+05	83.39%	6335	97.74	10.23
3.3	6.20E+05	1.45E+06	2.80	1.36E+06	28.97%	8.85E+05	65.22%	11045	170.40	5.87
4.1.1	6.20E+05	4.72E+06	4.75	4.42E+06	48.56%	1.89E+06	42.79%	14331	221.10	4.52
4.1.2	6.20E+05	2.29E+06	5.62	2.15E+06	58.09%	6.88E+05	32.06%	12582	194.11	5.15
4.2.1	6.20E+05	1.81E+06	3.31	1.67E+06	34.44%	9.93E+05	59.45%	10901	168.18	5.95
4.2.2	6.20E+05	1.86E+06	1.88	1.68E+06	39.15%	8.96E+05	53.26%	8503	131.18	7.62
4.3.1	6.20E+05	1.85E+06	4.16	1.75E+06	40.66%	8.94E+05	51.09%	7924	122.25	8.18
4.3.2	6.20E+05	1.59E+06	4.08	1.51E+06	37.14%	8.51E+05	56.49%	7667	118.29	8.45
5.1.1	6.20E+05	3.15E+05	0.76	2.99E+05	8.01%	2.70E+05	90.21%	4212	64.98	15.39
5.1.2	6.20E+05	4.79E+05	<0.2	4.51E+05	2.41%	4.35E+05	96.60%	4927	76.01	13.16
5.1.3	6.20E+05	6.14E+05	<0.2	5.79E+05	0.96%	5.70E+05	98.45%	4164	64.24	15.57
5.2.1	6.20E+05	6.32E+05	<0.2	6.02E+05	0.52%	5.94E+05	98.76%	4517	69.69	14.35
5.2.2	6.20E+05	5.78E+05	<0.2	5.47E+05	1.45%	5.33E+05	97.44%	4356	67.20	14.88
5.3.1	6.20E+05	7.88E+05	<0.2	7.48E+05	2.33%	7.21E+05	96.44%	7285	112.39	8.90
6.1.1	6.20E+05	1.55E+06	0.22	1.45E+06	3.63%	1.38E+06	94.90%	8777	135.41	7.38
6.1.2	6.20E+05	7.44E+05	0.27	6.97E+05	4.21%	6.57E+05	94.33%	7604	117.31	8.52
6.2.1	6.20E+05	7.47E+05	0.12	6.94E+05	2.93%	6.65E+05	95.81%	7771	119.89	8.34
6.2.2	6.20E+05	2.41E+06	1.45	2.14E+06	37.94%	1.10E+06	51.20%	8858	136.66	7.32
6.3.1	6.20E+05	7.81E+05	<0.2	7.43E+05	3.90%	7.04E+05	94.77%	3834	59.15	16.91
6.3.2	6.20E+05	1.17E+06	<0.2	1.12E+06	1.72%	1.09E+06	97.56%	3600	55.54	18.01
7.1.1	6.20E+05	3.12E+05	<0.2	2.97E+05	0.65%	2.92E+05	98.48%	4738	73.10	13.68
7.1.2	6.20E+05	5.97E+05	<0.2	5.63E+05	1.42%	5.49E+05	97.52%	5397	83.26	12.01
7.1.3	6.20E+05	1.52E+06	<0.2	1.44E+06	1.55%	1.41E+06	97.53%	8136	125.52	7.97
7.2.1	6.20E+05	2.70E+06	0.13	2.53E+06	2.38%	2.44E+06	96.48%	9081	140.10	7.14
7.2.2	6.20E+05	7.15E+05	0.27	6.73E+05	4.30%	6.34E+05	94.23%	7003	108.04	9.26
7.3.1	6.20E+05	8.22E+05	0.12	7.75E+05	1.96%	7.51E+05	96.92%	6943	107.12	9.34

Supplementary Table 3: List of strains, plasmids and primers used in this study

E. coli	**Relevent genotype/phenotype**	**Source/Reference**
EC100Dpir+	F– *mcrA* Δ(*mrr-hsdRMS-mcrBC*) ϕ80d*lacZ*ΔM15 Δ*lacX74 recA1 endA1 araD*139 Δ(ara, leu)7697 *galU galK* λ– *rpsL nupG pir*+ (DHFR)	#ECP09500 (Lucigen)
KN01	Sm^R Sp^R Nissle 1917	This study
KN01Rf^R	Spontaneous Rifiampicin resistant KN01	This study
KN01Δ*dapA*	Δ*dapA* KN01	This study
KN01ΔdapARf^R	Spontaneous Rifiampicin resistant KN01Δ*dapA*	This study
KN02	Sm^R Cm^R Nissle 1917	This study
KN02Nx^R	Spontaneous Nalidixic acid resistant KN02	This study
KN03	Sm^R Tc^R Nissle 1917	This study
MFD*pir*+	MG1655 RP4-2-Tc::[Δ*Mu1*::*aac*(3)IV-Δ*aphA*-Δ*nic35*-Δ*Mu2*::*zeo*] Δ*dapA*::(*erm-pir*) Δ*recA*	Ferrière *et al.* 2010[6]
MG1655Nx^R	K-12 *F– λ– ilvG– rfb-50 rph-1* Nx^R	Carraro *et al.* 2014[7]
Nissle 1917	Wildtype probiotic strain	DSM-6601 (DSMZ)

**Plasmid**	**Relevant genotype/phenotype**	**Source/Reference**
pBAD30	$oriV_{p15A}$, *bla, araC,* P_{BAD}	Guzman *et al.* 1995[5]
pE-FLP	$oriV_{pSC101ts}$, *flp*, Ap^R	Addgene #45978
pFG036	*cI*, *tetM*, *oriV* ColE1	Addgene #137996
pFG051	*aad7*, *cat*, Tn5 *tnp*, *oriV* R6K, *oriT* RP4	Addgene #137997
pGRG36	$oriV_{pSC101ts}$, Tn7 insertion machinery, *araC*, Ap^R	Addgene #16666
pGRG36-SmCm	pGRG36 with Sm^R Cm^R insert	This study
pGRG36-SmSp	pGRG36 with Sm^R Sp^R insert	This study
pGRG36-SmTc	pGRG36 with Sm^R Tc^R insert	This study
pKD3	R6K *oriV*, FRT flanked Cm^R, Ap^R	Addgene #45604
pKD4	R6K *oriV*, FRT flanked Km^R, Ap^R	Addgene #45605
pPilS	$oriV_{p15A}$, *cat, araC,* P_{BAD}, *pilS*	This study
pSB1C3	$oriV_{pMB1}$, *cat,* Biobricks	IGEM
pSIM6	$oriV_{pSC101ts}$, Lambda Red recombinase, Ap^R	Datsenko and Wanner 2000[8]
TP114Δ*pilS*::*cat*	TP114Δ*pilS*::*cat*	This study

**Primer**	**Sequence***	**Usage**
oGST1-F	GATCCTAGTAAGCCACGTTTTAATTAATCAGATCCCGGGCTAGTATGACGTCTGTCGCAC	*strA-strB* for pGRG36-SmTc
oGST1-R	GTAGGTTATTTATATAATTCATCCATTCCCATAACATCTGTTTACAGCTAGCTCAGTCCT	
oGST2-F	AAGCTAGCATAATACCTAGGACTGAGCTAGCTGTAAACAGATGTTATGGGAATGGATGAA	16S tag for pGRG36-SmTc
oGST2-R	CCCCAAAACTTTCCCCAAAACCCTTCCCCAAAACTGGCTATACTCGAGGCATGCCTGCAG	
oGST3-F	GGATTAGATACCCTGGTAGTCCTGCAGGCATGCCTCGAGTATAGCCAGTTTTGGGGAAGG	*tetB* for pGRG36-SmTc
oGST3-R	GGGGTCGACGCGGCCGTGGCGCGCCTCCTAGGTGCTCGAGCTAGGTCGACGCTTGGATTC	
oGSS1-F	GGATCCTAGTAAGCCACGTTTTAATTAATCAGATCCCGGGCCTACGGGAGGCAGCAGTGG	16S tag for pGRG36-SmSp
oGSS1-R	CGGGGAACTAGGAGGGTATGGTGCGCGCATGGAAAGACTACCAGGGTATCTAATCCTGTT	
oGSS2-F	CGCACCATACCCTCCTAGTTCCCCGGTTATCTCTCCTGTCTCTTATACACATCTGACGCT	*aad7* for pGRG36-SmSp
oGSS2-R	GGGGTCGACGCGGCCGTGGCGCGCCTCCTAGGTGCTCGAGGCAAGCGAACCGGAATTGCC	
oGSC1-F	GATCCTAGTAAGCCACGTTTTAATTAATCAGATCCCGGGCTAGTATGACGTCTGTCGCAC	*strA-strB* for pGRG36-SmCm
oGSC1-R1	TGCTAGCTTTGAAAATTAAGAGGTATATATTATTGAATCGAACTAATATTTTTTTTGGTG	
oGSC1-R2	GTGTCAACGTTTACAGCTAGCTCAGTCCTAGGTATTATGCTAGCTTTGAAAATTAAGAGG	Add P1-U8 for pGRG36-SmCm

oGSC2-F1	TACCTAGGACTGAGCTAGCTGTAAACGTTGACACCATCGAATGG**T**GCAAAACCTTTCGCG	*lacI-P$_{trc}$* for pGRG36-SmCm
oGSC2-F2	ATATCCCGAATGTGCAGTTAACGACGTTGACACCATCGAATGG**T**GCAAAACCTTTCGCGG	
oGSC2-R	TAATATATACCTCTTTAATTTTTAATAATAAAGTTAATCG	
oGSC3-F	TATTATTAAAAATTAAAGAGGTATATATTAATGGTTTCTAAAGGAGAAGAAAAAAATATG	NeonGreen for pGRG36-SmCm
oGSC3-R	CCTCTTTCCACTGCTGCCTCCCGTAGGTTATTTATATAATTCATCCATTCCCATAACATC	
oGSC4-F	AGCATTTACAGATGTTATGGGAATGGATGAATTATATAAATAACCTACGGGAGGCAGCAG	16S tag for pGRG36-SmCm
oGSC4-R	ACCTCTTACGTGCCCGATCAACTCGAGGCATGCCTGCAGGACTACCAGGGTATCTAATCC	
oGSC5-F	GAACAGGATTAGATACCCTGGTAGTCCTGCAGGCATGCCTCGAGTTGATCGGGCACGTAA	*cat f*or pGRG36-SmCm
oGSC5-R	GGTCGACGCGGCCGTGGCGCGCCTCCTAGGTGCTCGAGCTCGAGGCTTGGATTCTCACCA	
oGSC6-F	GAGCTTGGCGTAATCATGGTCATAGCTGTTTCCTGTGTGACGCTTAATGCGCCGCTACAG	Screen pGRG36 plasmid presence
oGSC6-R	CTGGTGAAAGTAAAAGATGCTGAAGATCAGTTGGGTGCACCGCGCAATTAACCCTCACTA	
oDTD1-F	CGCGTGCCTCGGCAAAATGCCCTTCTGCTGCCAGTTTGCAGTGTAGGCTGGAGCTGCTTC	*aph-IIIa* for *dapA* deletion
oDTD1-R	AAACGTACCATTGAGACACTTGTTTGCACAGAGGATGGCCCTGGGAATTAGCCATGGTCC	
oDTD2-F	CA*A*A*TAGTTTGTTGTGTAATGGCATCAGACGCTGATTAATAACGCGTGCCTCGGCAAAAT	Add homology
oDTD2-R	AC*G*G*TTCTGTCTGCTTGCTTTTAATGCCATACCAAACGTACCATTGAGACACTTGTTTGC	
oDTD3-int	CCGTTTCTGCGGACTGGCTT	Screen *dapA* deletion
oDTD23-F	TAGCGGCTATCACCAACATC	
oDTD3-R	GTGAAGCGCCTTATGAACAATG	
oTPS1-F	TGCTGAACCAGTAACAACCACC	TP114 1 bp sanger
oTPS1-R	GTCGCCGCTGTGGATTCAAC	
oTPS2-F	GTTCAATACACATTACAGCCCACC	TP114 10 kb sanger
oTPS2-R	CTGCGCTCAAAGTCACGTATGG	
oTPS3-F	TTACGCAACAGAATCTGAAAGCAC	TP114 20 kb sanger
oTPS3-R	GAAGGTGGCCTGTCATCGAG	
oTPS4-F	TGTCCGATTCGTCCTGGTTG	TP114 25 kb sanger
oTPS4-R	GTATTTGTCCAGCGCCCGG	
oTPS5-F	TTCAGATGCGTCGTGCAATG	TP114 37 kb sanger
oTPS5-R	CACACTTGAGCGTCTTTCTGA	
oTPS6-F	AGAAGCTCTTGAGTCCGACC	TP114 42,5 kb sanger
oTPS6-R	GACTTATTCCGCCAACCCAAATT	
oTPS7-F	GGCCCGCTCAAGGTCTTTC	TP114 47 kb sanger
oTPS7-R	GCTGGAGAACACCCTGATTATGT	
oTPS8-F	AAAGTTCTTTGCGCCTGTCATAGC	TP114 50 kb sanger
oTPS8-R	GAAGCCAGGTTTGTTGCTGTG	
oTPS9-F	TTTCTCTGCTACAGCATCTTTCTTC	TP114 52,5 kb sanger
oTPS9-R	GGAACTGCCTCGGTGAAT	
oTPS10-F	GGCATAAGGCGTGGACAATGG	TP114 57,5 kb sanger
oTPS10-R	CAAACGTGCTAATCGCCTGGC	
opilS1-F	ATGTCTTCTATTAATATTTTAAATATGCGTTCTGTTTTTTGTGTAGGCTGGAGCTGCTTC	Generate TP114Δ*pilS*::*cat*
opilS1-R	TCAGGAATCAGTGCTGAAGGTCAGCGTATTGCTGTCAGATATGGGAATTAGCCATGGTCC	
opilS2-F	TAACGTCCTGCAACACTAAT	Screen *pilS* deletion clones
opilS2-R	GCTTATCCGATGCACATGAA	

opPilS-F1	CTGTCAGACCAAGTTTACTC	*oriV*$_{p15A}$, *araC P$_{BAD}$* for pPilS
opPIlS-R1	TTTTGCCTCCTACGCTAGCCCAAAAAAACGGG	
opPilS-F2	AACCGTATTACCGCCTTTGAG	*cat* for pPilS
opPilS-R2	TCAATCTAAAGTATATATGAGTAAACTTGGTCTGACAGGTAAACTTGGTCTGACAGCTCG	
opPilS-F3	TCTCCATACCCGTTTTTTTGGGCTAGCGTAGGAGGCAAAAATGTCTTCTATTAATATTTT	*pilS* for pPilS
opPilS-R3	GGCGAGCGGTATCAGCTCACTCAAAGGCGGTAATACGGTTTCAGGAATCAGTGCTGAAGG	

Supplementary References:

1. Born, T. L. & Blanchard, J. S. Structure/function studies on enzymes in the diaminopimelate pathway of bacterial cell wall biosynthesis. *Curr. Opin. Chem. Biol.* **3**, 607–613 (1999).

2. St-Pierre, F. *et al.* One-step cloning and chromosomal integration of DNA. *ACS Synth. Biol.* **2**, 537–541 (2013).

3. Rodrigue, S. *et al.* Unlocking short read sequencing for metagenomics. *PLoS One* **5**, e11840 (2010).

4. Aziz, R. K. *et al.* The RAST Server : Rapid Annotations using Subsystems Technology. *BMC Genomics* **15**, 75–90 (2008).

5. Guzman, L., Belin, D. & Carson, M. J. Tight regulation , modulation , and high-level expression by vectors containing the arabinose P BAD promoter. *J. Bacteriol.* **177**, 4121–4130 (1995).

6. Ferrières, L. *et al.* Silent mischief: Bacteriophage Mu insertions contaminate products of *Escherichia coli* random mutagenesis performed using suicidal transposon delivery plasmids mobilized by broad-host-range RP4 conjugative machinery. *J. Bacteriol.* **192**, 6418–6427 (2010).

7. Carraro, N., Matteau, D., Luo, P. & Burrus, V. The master activator of IncA / C conjugative plasmids stimulates genomic islands and multidrug resistance dissemination. *PLoS Genet.* **10**, e1004714 (2014).

8. Datsenko, K. A. & Wanner, B. L. One-step inactivation of chromosomal genes in *Escherichia coli* K-12 using PCR products. *PNAS* **97**, 6640–6645 (2000).
