# Supplementary material for: Highly efficient gene transfer in the mouse gut microbiota is enabled by the Incl2 conjugative plasmid TP114
Source: Commun Biol. 2020 Sep 22;3:523. doi: 10.1038/s42003-020-01253-0 (PMC7508951; doi:10.1038/s42003-020-01253-0)
Supplement: Supplementary file 2 — Description of Additional Supplementary Items [file 42003_2020_1253_MOESM2_ESM.pdf]

Description of additional supplementary items

Supplementary Data 1: In silico analysis of gene function, essentiality, and conservation in conjugative plasmid TP114. The sequence of TP114 was annotated using the RAST server, and additional information about the function of predicted open reading frames (ORFs) was obtained using NCBI's BLAST and CDsearch tools. Gene essentiality was evaluated using transposon mutagenesis experiments presented in Figure 3.a (++, essential in all replicates; +, essential in at least one replicate; –, dispensable). Gene conservation in $IncI_2$ plasmids was derived from the analysis performed in Supplementary Figure 5, in which each gene was classified in the core (present in all tested IncI2 plasmids), soft core (present in >50% tested $IncI_2$ plasmids) or accessory (present in <50% of tested $IncI_2$ plasmids) category.

Supplementary Data 2: Source data for main figures 1 – 4.
